# Virologic and clinical characteristics of HBV genotypes/subgenotypes in 487 Chinese pediatric patients with CHB

**DOI:** 10.1186/1471-2334-11-262

**Published:** 2011-09-30

**Authors:** Yan-Wei Zhong, Jin Li, Hong-Bin Song, Zhong-Ping Duan, Yi Dong, Xiao-Yan Xing, Xiao-Dong Li, Mei-Lei Gu, Yu-Kun Han, Shi-Shu Zhu, Hong-Fei Zhang

**Affiliations:** 1 Pediatric Liver Disease Therapy and Research Center, Beijing 302 Hospital, Beijing, China; 2 Institute of Disease Control and Prevention, Academy of Military Medical Sciences, Beijing, China; 3 Beijing You'an Hospital, Capital Medical University, Beijing, China

**Keywords:** hepatitis B virus, genotypes/subgenoytpes, drug mutation, hepatic inflammation, fibrosis, pediatric patients

## Abstract

**Background:**

The association of hepatitis B virus (HBV) genotypes/subgenotypes with clinical characteristics is increasingly recognized. However, the virologic and clinical features of HBV genotypes/subgenotypes in pediatric patients remain largely unknown.

**Methods:**

Four hundred and eighty-seven pediatric inpatients with CHB were investigated, including 217 nucleos(t)ide analog-experienced patients. HBV genotypes/subgenotypes and reverse transcriptase (RT) mutations were determined by direct sequencing. The stage of fibrosis and degree of inflammatory activity were evaluated by the Metavir score system.

**Results:**

Among 487 enrolled pediatric patients, HBV genotype C2 and B2 were the most two prevalent (73.7% and 21.1%). Comparing with HBV/B2 infected patients, no significant difference was observed in the incidence rate and mutant patterns of lamivudine- or adefovir-resistant mutations in HBV/C2 infected patients (*P *> 0.05). Importantly, we found that the degree of hepatic inflammation degree, fibrosis stage and ALT level were significantly higher in HBV/C2-infected HBeAg positive patients than it was in HBV/B2-infected ones.

**Conclusions:**

The pediatric patients with HBV/C2 infection might be more susceptible to develop severe liver pathogenesis.

## Background

Hepatitis B virus (HBV) infection remains a serious health problem that approximately one third of the world's population has serological evidence of past or present infection with HBV and 350 million people are chronically infected. As a highly endemic area for HBV infection, it is estimated that 93 million people are infected with HBV in China [1-3]. Approximately 90% adults with chronic acquired the infection from infancy or in early childhood [4]. HBV as a highly variable virus is at least classified into eight genotypes based on a divergence in the entire nucleotide sequence greater than 8% [3-21]. Each HBV genotype can be further divided into subgenotypes on the basis of a greater than 4% but less than 8% divergence in the complete nucleotide sequence [3,12,14,22]. As of now, HBV/B, HBV/C, and HBV/D have been individually classified into five subgenotypes each, HBV/B1 to B5 [3,23], HBV/C1 to C5 [3,24,25] and HBV/D1 to D5 [3,24]. Some investigations indicated that C1 and C2 were predominant subgenotypes in the southern and northern parts of China, respectively [14,26-29]. Although the prevalence and clinical features of HBV genotypes/subgenotypes have been investigated in adult patients [3,9,30], However, the virologic and clinical features of HBV genotypes/subgenotypes in Chinese pediatric patients remain largely unknown.

## Methods

### Patients

Four hundred and eighty-seven pediatric inpatients with CHB who visited Beijing 302 Hospital from September 2007 to September 2010 were enrolled in the study. They were mainly from different regions of Northern China, All patients were anti-HCV and anti-HIV negative (375 males, 112 females, mean age 12 ± 5.7 years; range 3-18 years). The diagnostic and treatment criteria were based on "European Association for the Study of the Liver", 2009, EASL clinical practice guidelines: management of chronic hepatitis B [1]. For all patients, there was no evidence of HCC, concomitant of HCV, HDV, and HIV infection, metastatic or autoimmune liver disease. Two hundred and seventeen patients were exposed to nucleos(t)ide analog(s). The inclusion criteria were: all patients who were serum HBsAg positive for at least 6 months, but there was no evidence for HCC, or concomitant of HCV, HDV, HIV infection and autoimmune liver disease. The excluded criteria were: patients with acute hepatitis A, B, HCV, HDV, or HIV co-infection, and drug induced acute hepatitis, existence of renal failure, hepatic decompensation or psychiatric disorders, and central nervous system disease such as epilepsy, had received bone marrow or organ transplants, or had received immunosuppressive, nephrotoxic, or hepatotoxic medications within 2 months of enrollment. The study protocol was approved by the Beijing 302 Hospital Research Ethnics Committee, and written informed consents for therapy and study were obtained from each patient's parents.

### Detection of Serological Markers and Analysis of HBV Sequence

Serological markers and quantitation of HBV DNA, serum alanine aminotransferase (ALT), aspartate aminotransferase (AST), total bilirubin (TBIL), and other biochemical parameters were measured by standard procedures. HBeAg/anti-HBe, HBsAg/anti-HBs and anti-HBc were detected by enzyme-linked immunosorbent (Kewei Diagnostic Ltd., Beijing, China) or chemiluminescent assay (Abbott Laboratories, North Chicago, IL, USA). HBV DNA level was determined by a real-time polymerase chain reaction (PCR) kit (Fosun Pharmaceutical Co., Shanghai, China) with a lower limit of detection of 500 copies/mL (about 100 IU/mL). HBV genotype/subgenotype assignment was based on the analysis of the 1225 bp-long S/Pol-gene sequence (nt 54-1278) as described previously [31]. The sense and antisense primers designed respectively for the first-round PCR were desigened respectively followed [3]. Direct sequencing was performed using an ABI 3730xl DNA Analyzer (Applied Biosystems, Foster City, CA). Phylogenetic and molecular analyses were performed in MEGA version 4.0 [32]. Phylogenetic trees were constructed using neighbor-joining (NJ) analysis with bootstrap test confirmation performed on 1000 resampling standard reference sequences were acquired from the online Hepatitis Virus Database which can be found at: http://www.ncbi.nlm.nih.gov/projects/genotyping/formpage.cgi.

### Analysis of Genotypic/Subgenotypic Drug Mutations

Drug-resistance-associated mutations in the RT region of the HBV genome were analyzed as previously described [31]. Substitutions at positions rt80, rt173, rt180, rt181, rt204, rt214, rt229, rt233 and rt236 were taken as resistance-associated mutations for analysis.

### Analysis of Histopathological

The stage of fibrosis and the degree of inflammatory activity were evaluated by the Metavir score system [5], which classifies fibrosis into five stages: F0-no fibrosis, F1-portal fibrosis without septa, F2-portal fibrosis with few septa, F3-portal fibrosis with numerous septa, without cirrhosis, F4-cirrhosis. The degrees of inflammatory activity were divided into: A0-lack of histological activity, A1-mild histological activity, A2-moderate histological activity, A3-severe histological activity.

### Statistical Analysis

Measurement data were expressed as means ± standard deviations. Differences in Measurement data were examined by Student's *t *test and analysis of variance; the numeration data were analyzed by chi-square test. Logistic regression was used to evaluate *P *values in multivariate analysis. Statistical analysis was carried out with SPSS 16.0 software. A *P-*value of < 0.05 was considered statistically significant.

## Results

**Figure 1:**
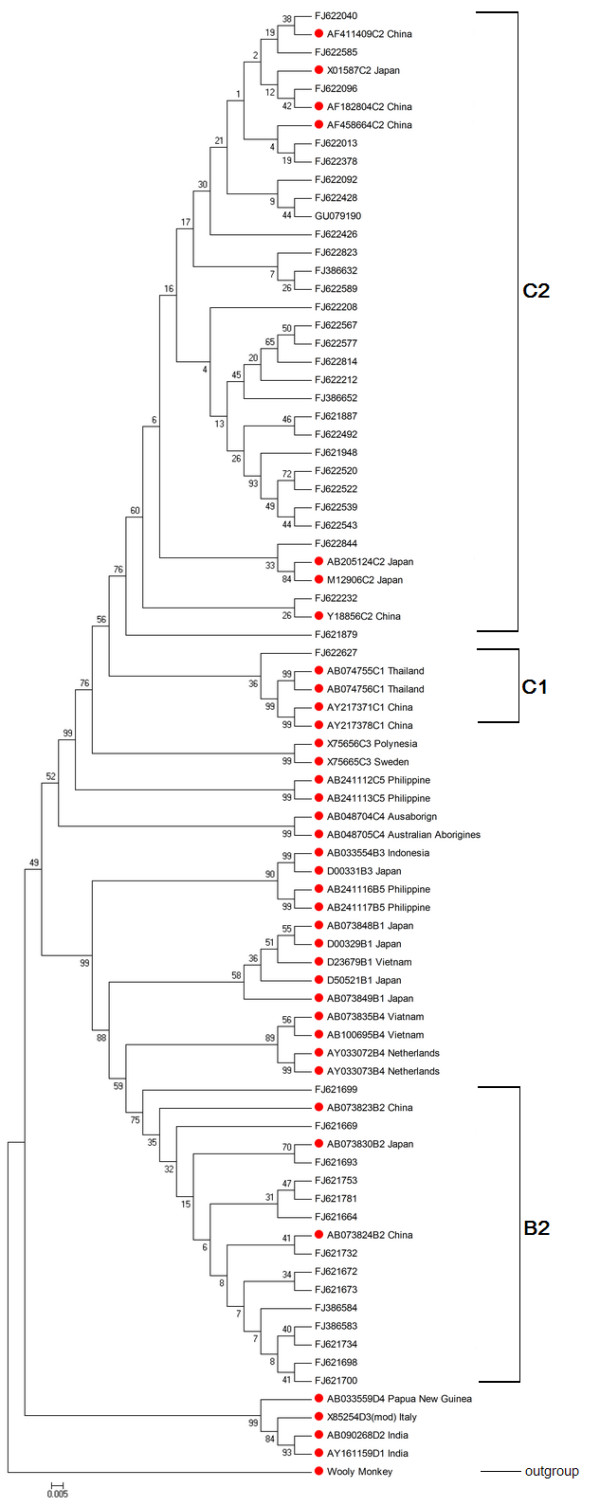
**Phylogenetic tree based on 43 representative HBV genetic sequences analyzed with GenBank accession numbers**. Standard reference sequences are marked by circles.

The main characteristics and the frequencies of the viral subgenotypes in the 487 pediatric patients are shown in Table 1. Among 487 enrolled pediatric patients, eighty-one percent of the patients were HBeAg positive (76.4% genotypes C; 22.3% B; 1.3% D), about 60% of them had alanine aminotransferase of up to 1.5 times the normal level (upper limit 40 U/L). As shown in Table 1, the subgenotype distribution was as follows: 1 (0.2%) for B1, 103 (21.1%) for B2 (for example: FJ621698, FJ621673, FJ621664, FJ621732, FJ621700, FJ621781, FJ621753, FJ621734, FJ621672, FJ621669, etc.), and 1 (0.2%) for B3, 3 (0.6%) for B4 (for example: FJ621693 and FJ621699), 7 (1.4%) for C1 (for example: FJ621879, FJ622823, etc.), 359 (73.7%) for C2 (for example: FJ622585, FJ386652, FJ622577, FJ621948, FJ386584, FJ622844, FJ386583, FJ622013, FJ386632, FJ622539, FJ622092, etc.), 5 (1.0%) for C3 (for example: FJ622096, FJ622627, etc.), 2 (0.4%) for C4, and 6 (1.2%) for D. No other genotypes (A, E, F, G, or H) were detected in enrolled samples enrolled for this study. Thus, HBV/C2 was the most predominant subgenotype among enrolled patients, followed by HBV/B2. A phylogenetic tree based on the 43 representative HBV genetic sequences, analyzed with GenBank accession numbers is presented in Figure 1. No obvious differences were observed in age, gender, serum HBV DNA level, TBIL, and HBeAg positive rate between HBV/C2 and HBV/B2 infected patients.

**Table 1:** Characteristics and HBV genotypes/subgenotypes frequencies among 487 pediatric patients

HBV subgenotype	B1	B2	B3	B4	C1	C2	C3	C4	D
Case number	1	103	1	3	7	359	5	2	6
Percentage (%)	0.2	21.1	0.2	0.6	1.4	73.7	1.03	0.41	1.23
Age (years)	13	15 ± 3	17	14 ± 3	16 ± 1	14 ± 3	14 ± 3	16 ± 1	14 ± 3
Male/female	1/0	81/22	1/0	3/0	6/1	275/84	2/3	2/0	4/2
HBV DNA (log IU/mL)	4.90	5.33 ± 1.63	5.35	5.56 ± 1.51	4.66 ± 1.68	5.50 ± 1.63	5.59 ± 0.91	4.98 ± 1.16	5.55 ± 2.09
TBIL (μmol/L)	10.9	11.48 ± 5.78	21.6	11.9 ± 6.65	9.54 ± 2.28	14.28 ± 34.93	7.9 ± 2.49	5.4 ± 2.8	10 ± 2.68
ALT (U/L)	10	70.48 ± 85.5	1002	179 ± 4.24	18 ± 8.28	116.8 ± 224.7	192.5 ± 166.0	29 ± 5.6	31.5 ± 2.12
HBeAg+ (n)	1	86	0	1	4	291	5	1	5

Among 217 nucleos(t)ide analog-experienced patients, a comparison of drug-resistant mutational patterns between HBV/B2 and HBV/C2 showed that the incidence rate and mutant patterns of LAM-resistant or ADV-resistant mutations were similar between the two subsets, and no significant difference was found (*P *> 0.05) (Tables 2 and 3).

**Table 2:** LAM-assosiated resistant mutantion in subgenotypes B2 and C2

Subgenotype	Total	M204I	M204V	M204I/V	L80I	V173L	L180M	M204I+CM
B2 (n = 30)	17 (56.7)	2 (11.7)	0	0	1 (5.8)	0	0	14 (82.3)
C2 (n = 76)	34 (44.7)	10 (29.4)	0	0	0	1 (2.9)	0	23 (67.6)
*P*	0.27	0.29	N	N	0.72	1	N	0.57

**Table 3:** ADV-assosiated resistant mutantion in subgenotypes B2 and C2

Subgenotype	Total	V84M	A181T/V	A181V+N236T	N236T	I233	V214A	L229V
B2 (n = 30)	5 (16.6)	0	1 (20.0)	1 (20.0)	0	0	0	3 (60.0)
C2 (n = 81)	19 (23.4)	0	4 (21.0)	0	0	4 (21.0)	4 (21.0)	7 (36.8)
*P*	0.44	N	1	0.208	N	0.544	0.544	0.615

Among the 487 pediatric inpatients with CHB, 187 HBeAg positive patients who were submitted to liver biopsy, the mean degree of inflammation was determined for HBV/C2 and HBV/B2 patients (2.1 ± 0.4 *vs *1.6 ± 0.4), and the mean stage of fibrosis was determined for HBV/C2 and HBV/B2 patients (1.9 ± 0.89 *vs *1.3 ± 0.6). The mean degree of inflammation, the stage of fibrosis and the ALT level in HBV/C2 patients were significantly higher than in HBV/B2 patients (*P *< 0.05) (Table 4). The sample size of HBeAg-negative patients was too small to be analyzed. Factors (such as sex, age, routes of infection, therapeutic effects) that can be affect the degree of inflammation, the stage of fibrosis and ALT level of HBV/B2, C2 patients have been analyzed by multiple logistic regression, and the results indicated that there is no statistically significant difference between them.

**Table 4:** The association of clinic character with HBV subgenotypes in HBeAg positive patients

Subgenotype	Inflammation degree	Fibrosis stage	DNA load (log IU/mL)	ALT (U/L)
B2 (n = 31)	1.6 ± 0.4	1.3 ± 0.6	7.7 ± 7.8	84 ± 87
C2 (n = 90)	2.1 ± 0.4	1.9 ± 0.8	7.8 ± 8.2	153 ± 194
*P*	0.002	0.039	0.545	0.047

## Discussion and conclusions

HBV genotypes/subgenotypes have been reported to have various effects on the clinical course of patients with HBV related liver diseases [2,4,7,9,13,18,21,33-37]. However, most of previous study data were from adult patients, studies on the pediatric patients were rare, especially in China. To our knowledge, there were seldom study to analyze the relationship among the genotypes/subgenotypes and NA resistance as well as the disease progression in pediatric patients. The relationship between HBV genotypes/subgenotypes and NA resistance, as well as the disease progression in pediatric patients, were analyzed in this study.

The data obtained from adult patients indicate that HBV genotypes/subgenotypes have distinct geographic distributions patterns [2,8,15,16,18,20-22,27,37-42]. Genotypes B and C are the most common HBV genotypes in China [14,28]. Our results indicate that among the 487 pediatric patients with CHB, HBV genotype C was the most prevalent (76.4%), while genotypes B and D were found in 22.3% and 1.3%, respectively. Genotypes E, F, G and H were not found. About 60% of these patients had ALT up to 1.5 times the normal level. The prevalence and the geographical pattern of the genotypes/subgenotypes of our pediatric patients were in consistent with previous reports in adults [3,41]. In addition, there were no significant genotypes/subgenotypes differences were found in age, gender, in serum HBV DNA levels, TBIL, and in HBeAg-positive rate among patients infected with HBV/B2 and HBV/C2.

NA are commonly used in clinical treatment for suppressing viral replication to halt the progression of liver diseases caused by chronic HBV infection [3,4,42,43]. The clinical implications of occurring HBV subgenotypes are far from well understood, especially in pediatric patients. Previous studies about adults exhibited that HBV/B2 differ from to HBV/C2 in LAM- or from ADV-resistant mutational patterns [3], suggesting that HBV subgenotypes might have an impact on drug resistance. However, when we compared the association of LAM- or ADV- resistance mutational rates with HBV/B2 and HBV/C2 in pediatric patients respectively, we did not find significant differences between the two groups. The difference between adults and pediatric patients in this respect may be explained by following reasons: (1) adult patients possibly have a longer time of infection and therapy than do pediatric patients, which may increase the incidence rate and extent of NA-related resistance mutations. (2) The different host's immune response to the virus between the adults and pediatric patients determines the extent of NA resistant rates and patterns [2]. Additional large population-based studies are needed to confirm this inference.

In this study, we also analyzed the relationship between subgenotypes and disease progression. We found that HBV/B2 patients had a significantly lower incidence of inflammation and fibrosis than HBV/C2 in pediatric patients. Moreover, in our previous studies, the double mutation in BCP (T1762/A1764) was obviously more frequent in genotype C (26%) than that in genotype B patients (9.8%, *P *< 0.05). However, there was no significant difference in preC (A1896) between genotype B (3.7%) and genotype C (2.8%, *P *> 0.05). According to the relevant literature, there are some correlations between the mutation in HBV BCP region and disease progression [44].

In conclusion, HBV/C2 is the most predominant subgenotype in pediatric patients of Northern China. Pediatric patients with subgenotype C2 virus might be more susceptible to disease progression. Nucleos(t)ide analog therapeutic decisions might not be based primarily on genotypes/subgenotypes in pediatric patients at present. Our results provide new insights in features of HBV subgenotypes which may have important clinical implications for the management of HBV infections in pediatric patients.

## Competing interests

The authors declare that they have no competing interests.

## Authors' contributions

YWZ, HFZ, SSZ designed the study. YWZ, YD, XYX, XDL, MLG, YKH performed experiments. YWZ, JL, HBS, ZPD analyzed data and discussed results. YWZ, SSZ, HFZ wrote manuscript. All authors read and approved the final manuscript.

## Pre-publication history

The pre-publication history for this paper can be accessed here:

http://www.biomedcentral.com/1471-2334/11/262/prepub
